# Conflict between Translation Initiation and Elongation in Vertebrate Mitochondrial Genomes

**DOI:** 10.1371/journal.pone.0000227

**Published:** 2007-02-21

**Authors:** Xuhua Xia, Huang Huang, Malisa Carullo, Esther Betrán, Etsuko N. Moriyama

**Affiliations:** 1 Department of Biology, University of Ottawa, Ottawa, Ontario, Canada; 2 Center for Advanced Research in Environmental Genomics, University of Ottawa, Ottawa, Ontario, Canada; 3 Ottawa Institute of Systems Biology, University of Ottawa, Ottawa, Canada; 4 Department of Biology, University of Texas at Arlington, Arlington, Texas, United States of America; 5 School of Biological Sciences and Plant Science Institute, University of Nebraska-Lincoln, Lincoln, Nebraska, United States of America; Wellcome Trust Centre for Human Genetics, United Kingdom

## Abstract

The strand-biased mutation spectrum in vertebrate mitochondrial genomes results in an AC-rich L-strand and a GT-rich H-strand. Because the L-strand is the sense strand of 12 protein-coding genes out of the 13, the third codon position is overall strongly AC-biased. The wobble site of the anticodon of the 22 mitochondrial tRNAs is either U or G to pair with the most abundant synonymous codon, with only one exception. The wobble site of Met-tRNA is C instead of U, forming the Watson-Crick match with AUG instead of AUA, the latter being much more frequent than the former. This has been attributed to a compromise between translation initiation and elongation; i.e., AUG is not only a methionine codon, but also an initiation codon, and an anticodon matching AUG will increase the initiation rate. However, such an anticodon would impose selection against the use of AUA codons because AUA needs to be wobble-translated. According to this translation conflict hypothesis, AUA should be used relatively less frequently compared to UUA in the UUR codon family. A comprehensive analysis of mitochondrial genomes from a variety of vertebrate species revealed a general deficiency of AUA codons relative to UUA codons. In contrast, urochordate mitochondrial genomes with two tRNA^Met^ genes with CAU and UAU anticodons exhibit increased AUA codon usage. Furthermore, six bivalve mitochondrial genomes with both of their tRNA-Met genes with a CAU anticodon have reduced AUA usage relative to three other bivalve mitochondrial genomes with one of their two tRNA-Met genes having a CAU anticodon and the other having a UAU anticodon. We conclude that the translation conflict hypothesis is empirically supported, and our results highlight the fine details of selection in shaping molecular evolution.

## Introduction

Vertebrate mitochondrial DNA has two strands of different buoyant densities, i.e., the H-strand and the L-strand. The H-strand is the sense strand for 1 protein-coding gene (ND6) and 8 tRNA genes and the L-strand is the sense strand for 12 protein-coding genes, 2 rRNA genes and 14 tRNA genes. The two strands have different nucleotide frequencies, with the H-strand being GT-rich and the L-strand AC-rich [1], [2]. This asymmetrical distribution of nucleotides has been explained [3]–[6] in terms of the strand-displacement model of mitochondrial DNA (mtDNA) replication [7]–[10]. In short, the H-strand is left single-stranded for an extended period and subject to spontaneous deamination of A and C [11], [12] to G and U. In particular, the C→U mutation mediated by the spontaneous deamination is known to occur in single-stranded DNA about 100 times as frequently as in double-stranded DNA [13]. Therefore, the H-strand tends to accumulate A→G and C→U mutations and become GT-rich while the L-strand tends to become AC-rich. This pattern is similar to the strand bias observed in eubacterial genomes [14]–[16].

The strand-biased mutation spectrum has profound consequences on codon usage in mitochondrial protein-coding sequences (CDSs) and the anticodon of tRNA genes [17]. First, the codons of the 12 CDS sequences (that are collinear with the AC-rich L-strand) end mainly with A or C, and the codon bias in the ND6 gene collinear with the opposite strand is the opposite. Second, the 8 tRNA sequences collinear with the GT-rich H-strand is more GT-rich than the 14 tRNA sequences collinear with the AC-rich L-strand. Third, because the overall codon usage is mainly determined by the 12 CDSs collinear with the AC-rich L strand, the A-ending and C-ending codons are almost always the most frequently used codons. The anticodon of 21 tRNA genes (out of a total of 22), regardless of which strand they are located, have anticodons with their wobble site forming Watson-Crick base-paring with the most abundant codons in each codon family, i.e., the wobble site of the tRNA genes is either a U to pair with the abundant A-ending codons or a G to pair with the abundant C-ending codons [17].

The codon-anticodon adaptation is long known [18]–[23], and the pattern described above would have been nice but boring had there not been an interesting and singular exception to the general pattern of tRNA anticodon matching the most abundant codon. The tRNA^Met^ anticodon is 3′-UAC-5′ (or CAU for short), with the wobble site being C instead of U, and forms a Watson-Crick match with the AUG codon instead of the AUA codon, in spite of the fact that the latter is used much more frequently than the former. The ability of the CAU anticodon to pair with the AUA codon is achieved by modifying the C in the anticodon CAU to 5-formylcytidine [24], [25]. A similar case involves the methylation of guanine in starfish tRNA^Ser^ to translate all four AGN codons [25].

The use of the CAU anticodon instead of a UAU anticodon in vertebrate mitochondrial tRNA^Met^ is unexpected from two existing hypotheses of anticodon usage. The codon-anticodon adaptation hypothesis [17]–[20], [23], [26] predicts that the anticodon should match the most abundant codon. Because AUA is much more frequent than AUG, the hypothesis predicts that the anticodon of the tRNA^Met^ gene should be UAU instead of the observed CAU. The hypothesis of selection on anticodon wobble versatility [17], which was implicitly proposed before [27], [28] and may be more appropriate for vertebrate mitochondrial genomes because each codon family is translated by a single tRNA species, states that the anticodon should maximize its wobble versatility in paring with synonymous codons. Because U in general is more versatile than C in wobble pairing with both A and G [29], [30], the hypothesis of selection on anticodon versatility also predicts an UAU anticodon to maximize its paring versatility with the AUA and AUG codons. The fact that the observed tRNA^Met^ anticodon is CAU instead of the predicted UAU is intriguing.

This unexpected tRNA^Met^ anticodon has been attributed to a compromise between translation initiation and elongation [17] as follows. AUG is not only the most frequently used initiation codon, but also the most efficient initiation codon in *Escherichia coli* [31] and *Saccharomyces cerevisiae* [32]. In *E. coli*, the most efficient non-AUG initiation codon is AUA and its rate of initiation is only 7.5% of AUG [31]. In yeast mitochondria, a mutation of the initiation AUG to AUA in the COX2 gene caused at least a five-fold decrease in translation [33], and similar finding was also duplicated in another yeast mitochondrial gene COX3 [34]. Assuming the generality of these findings, an anticodon matching AUG will increase the initiation rate and would be favored by natural selection because translation initiation is often the limiting step in protein production [22], [35]. This presents a conflict between translation initiation and translation elongation. An AUG-matching anticodon would increase the translation initiation rate but decrease the translation elongation rate because an overwhelming majority of methionine codons are AUA in vertebrate mitochondrial genomes. The fact that all known vertebrate tRNA^Met^ genes feature an AUG-matching anticodon implies that nature has chosen to maximize the translation initiation rate [17]. This hypothesis that invokes a conflict between translation initiation and translation elongation to explain the usage of the CAU anticodon in tRNA^Met^ will be referred hereafter as the translation conflict hypothesis.

Two consequences can be derived from the translation conflict hypothesis. First, we should expect a relative reduction of AUA usage because the AUG-matching anticodon imposes selection against the use of AUA codons as AUA would need to be wobble-translated. To fix ideas, let us focus only on AUR (methionine) and UUR (leucine) codon families. The reason for choosing UUR instead of any other R-ending codon families is because other R-ending codon families do not have a middle U and the middle nucleotide in a codon is known to affect the nucleotide at the third codon position (P. Higgs, pers. comm.).

For the 12 CDSs that are collinear with the AC-rich L-strand, the mutation favors A-ending codon [3], [4], [17]. For UUR codons, because the anticodon wobble site is U and form Watson-Crick base pair with A, we also expect UUA codon to be preferred against UUG codons. Thus, both mutation and the tRNA-mediated selection favor the use of UUA against UUG codons. However, for the methionine codons, the AUG-matching tRNA^Met^ anticodon would favor the AUG codon against the AUA codon. Thus, the tRNA-mediated selection and the mutation bias are in opposite directions. If we define 
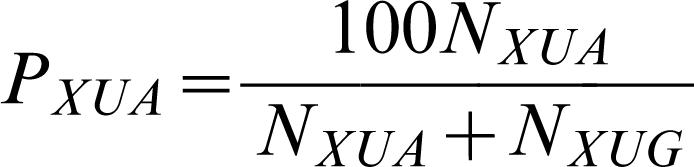
1for each of these two codon families, where X is either A or U, and N_XUA_ and N_XUG_ are the number of XUA and XUG codons, respectively, we should find P_AUA_ to be smaller in the AUR codon family than P_UUA_ in the UUR codon family.

An argument against using Eq. (1) is that the result would be biased in favor of supporting the prediction of P_AUA_<P_UUA_ because the initiation codon, which is AUG in most cases, was not excluded. A more convincing comparison should compute P_AUA_ after excluding initiation codons entirely. This is what we used in this study.

For the ND6 gene collinear with the GT-rich H-strand, the strand-biased mutation spectrum favors G-ending codons in the two XUR codon families. For the methionine codon family, the AUG-matching anticodon also favors the AUG codon against the AUA codon. So the AUA codon will be depressed by both the strand-biased mutation and the tRNA-imposed selection. The tRNA-imposed selection is absent against UUA codon in the UUR codon families because their respective tRNA anticodons all match the A-ending codons [17]. Thus, for the ND6 gene, we also expect P_AUA_ to be smaller in the AUR codon family than P_UUA_ in the UUR codon family.

The expected P_AUA_<P_UUA_, if confirmed, can have two possibilities. If the total number of methionine remains constant across mitochondrial genomes, then a deficiency of the AUA codons in one genome implies an equal amount of surplus of AUG codons. In contrast, if there is no selection maintaining a constant number of methionine codons but there is selection against AUA codons because it requires the inefficient wobble translation, then a genome with a deficiency of AUA codons would also exhibit a deficiency of methionine codons.

In this paper, we use mitochondrial genomes from representative vertebrates, urochordates and bivalves to test these predictions (with the relevance of the bivalve mitochondrial genomes pointed out to us by an anonymous reviewer). While vertebrate mitochondrial genomes all have just one tRNA-Met gene, urochordates have two tRNA-Met genes, with one having a CAU anticodon and the other having an UAU anticodon. The presence of the UAU anticodon in the tRNA-Met gene in urochordate mitochondrial genomes implies that the selection against AUA codon should be weaker in urochordates than in vertebrates. The bivalve mitochondrial genomes are particularly interesting because some of them have anticodon CAU in both of their tRNA^Met^ genes whereas others have an anticodon CAU in one of their tRNA^Met^ genes and an anticodon UAU in the other tRNA^Met^ gene. We expect the selection against the AUA codon to be weaker in the latter than in the former.

## Materials and Methods

To test the predictions derived from the translation conflict hypothesis, we retrieved all 498 vertebrate mitochondrial genomes available from NCBI Entrez by Sept. 29, 2005. The CDS sequences from each mitochondrial genome were extracted and codon usage quantified by using DAMBE [36], [37], and separated into two groups, with one containing the 12 CDSs collinear with the L-strand and the other containing the ND6 gene collinear with the H-strand. We tabulated the frequencies of the A-ending and G-ending codons for AUR and UUR codon families for each of the two groups. The CDS-derived amino acid usage is also computed with DAMBE.

P_XUA_ values as defined in Eq. (1) are computed for each of the six codon families, with the initiation codon excluded. Many of these mitochondrial genomes are very similar to each other and detailed analysis were carried out for 30 species, with six species each from teleosts, amphibians, non-avian sauropods, birds, and mammals. The reason for choosing 30 species is that a random selection of any 30 species, with six from each of the five groups, always lead to the same conclusion based on multiple random selections. The 30 species is just one of many samples of 30 species.

It is important to keep in mind that the 30 species above do not represent independent data points. For example, their common ancestor could have somehow evolved a reduced P_AUA_ relative to P_UUA_, and this character has been inherited among all its descendents. This means that all 30 species could be equivalent to just single data point. For this reason, corroborative evidence needs to be sought in other species.

The mitochondrial genomes of four urochordates (*Halocynthia roretzi, Ciona intestinalis, C. savignyi*, and *Doliolum nationalis*) deposited in GenBank are particularly relevant to this study for several reasons. First, all 13 protein-coding genes are located in one strand, which eliminates the effect of differential strand-biased mutation on codon usage, i.e., all genes are subject to the same strand-specific mutation bias, if any. Second, they all have two tRNA^Met^ genes, one with a CAU anticodon and the other with a UAU anticodon [38]–[43]. This would eliminate, or at least reduce, the hypothesized selection against AUA codon usage. We can therefore predict that P_AUA_ should be increased relative to P_UUA_ in these urochordate mitochondrial genomes compared to vertebrate mitochondrial genomes.

One anonymous reviewer pointed out to us that another contrast can be made within mitochondrial genomes of bivalve mollusks. Ten complete bivalve mitochondrial genomes are available in GenBank. Aside from *Lampsilis ornata* which has undergone a great deal of genome rearrangement with protein-coding genes distributed on both strands and has only one tRNA^Met^ gene [44], the other nine bivalve species all have two tRNA^Met^ genes in their mitochondrial genomes and all have their protein-coding genes in the same strand. Among these nine species, six of them have two CAU-tRNA^Met^ gene (matching the AUG codon), whereas the other three has a CAU-tRNA^Met^ gene and a UAU-tRNA^Met^ gene (matching the AUA codon) in the other tRNA-Met gene. We predict that tRNA-mediated selection against the AUA codon should be stronger in the former than in the latter according to the translation conflict hypothesis.

## Results and Discussion

For the 30 vertebrate mitochondrial genomes, the mean P_AUA_ value is consistently smaller than the mean P_UUA_ values (Table 1), consistent with the prediction from the translation conflict hypothesis. The prediction is strongly supported by data from both the 12 CDSs collinear with the L-strand and the ND6 gene collinear with the H strand (Table 1).

**Table 1:** Results from the 12 CDSs collinear with the L-strand and ND6 collinear with the H-strand, with results from individual paired-sample t-tests between P_UUA_ and P_AUA_.

		12 CDS		ND6	
Species	Accession	P_UUA_	P_AUA_	P_UUA_	P_AUA_
*Homo sapien*	NC_001807	87.8	87.1	53.3	22.2
*Mus musculus*	NC_005089	94.2	93.4	65.2	40.0
*Bos taurus*	NC_006853	90.1	87.8	62.5	40.0
*Canis familiaris*	NC_002008	85.3	85.5	43.8	50.0
*Equus caballus*	NC_001640	86.4	87.5	25.0	11.1
*Capra hircus*	NC_005044	94.4	88.5	68.8	66.7
*Gallus gallus*	NC_001323	94.9	88.2	31.6	25.0
*Struthio camelus*	NC_002785	89.2	87.9	27.3	0.0
*Coturnix chinensis*	NC_004575	95.7	88.0	28.6	0.0
*Anser albifrons*	NC_004539	92.2	82.9	41.7	0.0
*Gavia stellata*	NC_007007	88.0	91.9	7.1	33.3
*Alectura lathami*	NC_007227	83.1	80.4	61.1	25.0
*Alligator mississippiensis*	NC_001922	94.2	84.4	64.7	33.3
*Alligator sinensis*	NC_004448	78.2	76.1	45.5	37.5
*Chelonia mydas*	NC_000886	99.1	98.6	30.4	75.0
*Shinisaurus crocodilurus*	NC_005959	94.2	89.0	42.9	33.3
*Abronia graminea*	NC_005958	92.0	91.2	42.9	12.5
*Chrysemy picta*	NC_002073	92.7	94.5	50.0	33.3
*Ambystoma laterale*	NC_006330	95.0	91.5	91.3	71.4
*Aneides hardii*	NC_006338	92.6	85.4	45.0	77.8
*Xenopus laevis*	NC_001573	93.6	88.6	64.7	75.0
*Kaloula pulchra*	NC_006405	87.9	76.7	47.8	0.0
*Alytes obstetricans*	NC_006688	90.5	80.1	57.1	0.0
*Rana nigromaculata*	NC_002805	91.3	69.1	66.7	40.0
*Cyprinus carpio*	NC_001606	97.7	79.1	62.5	20.0
*Danio rerio*	NC_002333	89.6	78.9	57.1	50.0
*Salanx ariakensis*	NC_006918	73.9	46.9	77.8	0.0
*Carassius auratus*	NC_002079	93.9	75.5	60.0	40.0
*Anguilla rostrata*	NC_006547	89.1	83.2	57.9	66.7
*Auxis rochei*	NC_005313	82.8	38.8	83.3	12.5
Mean		90.3	82.6	52.1	33.1
T		4.2591		3.7612	
P (1-tailed)		0.0001		0.0004	

**Figure 1:**
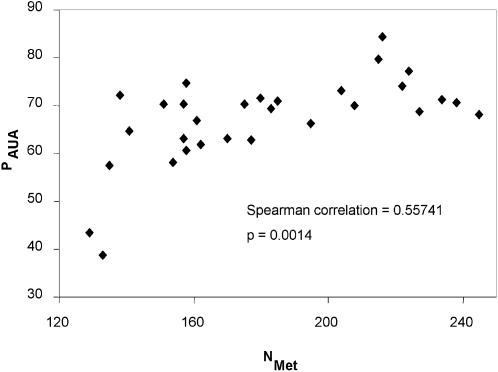
Genomic reduction of AUA codons is associated with a reduction in methionine usage. P_AUA_ is defined in equation (1) and arcscine-transformed; N_Met_ – Number of methionine codons.

The observation of a relative deficiency of AUA codons can be interpreted in two ways. If methionine usage remains constant among vertebrate mitochondrial genomes, then a deficiency of AUA in a genome implies an equal amount of surplus in AUG. On the other hand, if the number of methionine codons (N_Met_) is weakly constrained, then the selection against AUA codons may result in a net loss of methionine codons. This would lead to a positive association between P_AUA_ and N_Met_, i.e., small P_AUA_ is associated with small N_Met_. The empirical data supports the latter inference, i.e., reduction of AUA codons leads to a reduction in methionine usage in the genome (Fig. 1).

It is important to recognize that the results presented above, while all consistent with the translation conflict hypothesis, do not exclude the possibility that AUA codon usage may be reduced for reasons unrelated to the CAU anticodon in tRNA^Met^. It would be nice to have a mitochondrial genome in which the tRNA^Met^ anticodon is not CAU but UAU. If such a genome also has a reduced AUA usage relative to UUA codons, then we cannot interpret the reduced AUA usage in the vertebrate mitochondrial genomes as a response to the selection mediated by the CAU anticodon in tRNA^Met^. On the other hand, if such a genome does not exhibit a deficiency of AUA codons relative to UUA codons, but instead exhibit an increased AUA codon usage favored by the UAU anticodon, then the translation conflict hypothesis is strengthened.

In this context the mitochondrial genomes of four urochordates (*Halocynthia roretzi, Ciona intestinalis, C. savignyi*, and *Doliolum nationalis*) deposited in GenBank are particularly useful in providing corroborative evidence. All four genomes have two tRNA^Met^ genes, one with CAU anticodon and the other with a UAU anticodon [38]–[43]. This would eliminate, or at least reduce, the hypothesized selection against AUA codon usage. We can therefore predict that P_AUA_ should be increased relative to P_UAU_ for these urochordate genomes, in contrast to the vertebrate mitochondrial genomes in which the only tRNA^Met^ has a CAU anticodon that would favor a decreased usage of AUA codons. In other words, we should expect (*P_UUA_−P_AUA_*) to smaller in the urochordate mitochondrial genomes than (*P_UUA_−P_AUA_*) in vertebrate mitochondrial genomes. This prediction is confirmed (Table 2). The mean (*P_UUA_−P_AUA_*) is only −3.225 in the urochordate mitochondrial genomes, in contrast to the vertebrate mitochondrial genomes where the mean (*P_UUA_−P_AUA_*) values are significantly greater than 0 (p = 0.0001 for the 12 CDSs collinear with the L-stand and p = 0.0004 for ND6 collinear with the H-strand).

**Table 2:** Results from the 13 CDSs from the four urochordate species, *Halocynthia roretzi, Ciona intestinalis, Ciona savignyi*, and *Doliolum nationalis*, whose mitochondrial genomes each have a UAU-tRNA^Met^ gene in addition to a CAU-tRNA^Met^ gene.

Species	Accession	P_UUA_	P_AUA_	P_UUA_−P_AUA_
*H. roretzi*	NC_002177	60.7	67.3	−6.6
*C. intestinalis*	NC_004447	92.6	90.5	2.1
*C. savignyi*	NC_004570	75.1	83.5	−8.4
*D. nationalis*	NC_006627	71.0	71.0	0.0

Another independent contrast can be made within bivalve mollusks. Ten bivalve mitochondrial genomes are publicly available in GenBank. Aside from *Lampsilis ornata* which differs from the other species in that (1) it has undergone a great deal of genome rearrangement with protein-coding genes distributed on both strands and (2) has only one tRNA gene for methionine [44], the other nine species all have two tRNA^Met^ genes and all have their protein-coding genes in the same strand. Among these nine species, six of them have two CAU-tRNA^Met^ genes (matching AUG codon), whereas the other three has one CAU-tRNA^Met^ gene and one UAU-tRNA^Met^ gene (matching AUA codon). We have predicted that tRNA-mediated selection against AUA codon should be stronger in the former than in the latter according to the translation conflict hypothesis. The prediction is strongly supported (Table 3). The six genomes with the two CAU-tRNA^Met^ genes, labeled CAU/CAU, have P_UUA_ greater than P_AUA_, but the three genomes with one CAU-tRNA^Met^ gene and one UAU-tRNA^Met^ gene, labelled CAU/UAU, have P_UUA_ smaller than P_AUA_ (Table 3). We did not perform a significance test between the two groups of species because the three species with CAU/UAU anticodons are all in the *Mytilus* genus. So the contrast should be taken as only one contrast.

**Table 3:** The effect of anticodons (AC) of the two tRNA-Met genes in bivalve mitochondrial genomes on P_UUA_ and P_AUA_.

Species	Accession	AC	P_UUA_	P_AUA_	P_UUA_−P_AUA_
Acanthocardia tuberculata	NC_008452	CAU/CAU	65.65	56.28	9.36
Hiatella arctica	NC_008451	CAU/CAU	73.96	60.34	13.61
Crassostrea virginica	NC_007175	CAU/CAU	63.18	47.34	15.84
C. gigas	NC_001276	CAU/CAU	64.83	46.89	17.94
V.philippinarum	NC_003354	CAU/CAU	80.18	74.07	6.10
Placopecten magellanicus	NC_007234	CAU/CAU	37.50	33.99	3.51
Mytilus trossulus	NC_007687	CAU/UAU	58.66	60.61	−1.94
M. galloprovincialis	NC_006886	CAU/UAU	63.83	65.02	−1.19
M. edulis	NC_006161	CAU/UAU	63.12	65.30	−2.18

Those with only CAU-tRNA^Met^ genes have reduced AUA usage than those with both CAU-tRNA^Met^ and UAU-tRNA-^Met^ genes.

In conclusion, the translation conflict hypothesis is empirically supported. The presence of a CAU anticodon matching the AUG methionine codon represents a significant selection force against AUA codon usage in vertebrate mitochondrial genomes, resulting in *P_AUA_* smaller than *P_UUA_*. The reduced AUA codon usage is associated with a reduced methionine usage in the vertebrate mitochondrial genomes. When such selection is weakened in the urochordate mitochondrial genomes containing CAU-tRNA^Met^ and UAU-tRNA^Met^ genes, the AUA codon is no longer strongly selected against, and *P_AUA_* becomes similar to *P_UUA_*. In bivalve mollusks, mitochondrial genomes with only CAU-tRNA^Met^ genes have reduced AUA usage than those with both CAU-tRNA^Met^ and UAU-tRNA-^Met^ genes.

## References

[1] Perna NT, Kocher TD (1995). Patterns of nucleotide composition at fourfold degenerate sites of animal mitochondrial genomes.. J Mol Evol.

[2] Jermiin L, Graur D, Crozier R (1995). Evidence from Analyses of Intergenic Regions for Strand-specific Directional Mutation Pressure in Metazoan Mitochondrial DNA.. Molecular Biology and Evolution.

[3] Reyes A, Gissi C, Pesole G, Saccone C (1998). Asymmetrical directional mutation pressure in the mitochondrial genome of mammals.. Mol Biol Evol.

[4] Tanaka M, Ozawa T (1994). Strand asymmetry in human mitochondrial DNA mutations.. Genomics.

[5] Faith JJ, Pollock DD (2003). Likelihood analysis of asymmetrical mutation bias gradients in vertebrate mitochondrial genomes.. Genetics.

[6] Raina SZ, Faith JJ, Disotell TR, Seligmann H, Stewart CB (2005). Evolution of base-substitution gradients in primate mitochondrial genomes.. Genome Res.

[7] Clayton DA (1982). Replication of animal mitochondrial DNA.. Cell.

[8] Shadel GS, Clayton DA (1997). Mitochondrial DNA maintenance in vertebrates.. Annu Rev Biochem.

[9] Clayton DA (2000). Transcription and replication of mitochondrial DNA.. Hum Reprod.

[10] Bogenhagen DF, Clayton DA (2003). The mitochondrial DNA replication bubble has not burst.. Trends Biochem Sci.

[11] Sancar A, Sancar GB (1988). DNA repair enzymes.. Annual Review of Biochemistry.

[12] Lindahl T (1993). Instability and decay of the primary structure of DNA.. Nature.

[13] Frederico LA, Kunkel TA, Shaw BR (1990). A sensitive genetic assay for the detection of cytosine deamination: determination of rate constants and the activation energy.. Biochemistry.

[14] McInerney JO (1998). Replicational and transcriptional selection on codon usage in Borrelia burgdorferi.. Proc Natl Acad Sci U S A.

[15] Lobry JR (1996). Asymmetric substitution patterns in the two DNA strands of bacteria.. Mol Biol Evol.

[16] Lobry JR, Sueoka N (2002). Asymmetric directional mutation pressures in bacteria.. Genome Biol.

[17] Xia X (2005). Mutation and selection on the anticodon of tRNA genes in vertebrate mitochondrial genomes.. Gene.

[18] Ikemura T (1981). Correlation between the abundance of *Escherichia coli* transfer RNAs and the occurrence of the respective codons in its protein genes: a proposal for a synonymous codon choice that is optimal for the E coli translational system.. J Mol Biol.

[19] Gouy M, Gautier C (1982). Codon usage in bacteria: correlation with gene expressivity.. Nucleic Acids Res.

[20] Bennetzen JL, Hall BD (1982). Codon selection in yeast.. J Biol Chem.

[21] Bulmer M (1987). Coevolution of codon usage and transfer RNA abundance.. Nature.

[22] Bulmer M (1991). The selection-mutation-drift theory of synonymous codon usage.. Genetics.

[23] Xia X (1998). How optimized is the translational machinery in Escherichia coli, Salmonella typhimurium and Saccharomyces cerevisiae?. Genetics.

[24] Moriya J, Yokogawa T, Wakita K, Ueda T, Nishikawa K (1994). A novel modified nucleoside found at the first position of the anticodon of methionine tRNA from bovine liver mitochondria.. Biochemistry.

[25] Matsuyama S, Ueda T, Crain PF, McCloskey JA, Watanabe K (1998). A novel wobble rule found in starfish mitochondria. Presence of 7-methylguanosine at the anticodon wobble position expands decoding capability of tRNA.. J Biol Chem.

[26] Ikemura T, Hatfield DL, Lee B, Pirtle J (1992). Correlation between codon usage and tRNA content in microorganisms.. Transfer RNA in protein synthesis..

[27] Heckman JE, Sarnoff J, Alzner-DeWeerd B, Yin S, RajBhandary UL (1980). Novel features in the genetic code and codon reading patterns in Neurospora crassa mitochondria based on sequences of six mitochondrial tRNAs.. Proc Natl Acad Sci U S A.

[28] Martin RP, Sibler AP, Gehrke CW, Kuo K, Edmonds CG (1990). 5-[[(carboxymethyl)amino]methyl]uridine is found in the anticodon of yeast mitochondrial tRNAs recognizing two-codon families ending in a purine.. Biochemistry.

[29] Tong KL, Wong JT (2004). Anticodon and wobble evolution.. Gene.

[30] Agris PF (2004). Decoding the genome: a modified view.. Nucleic Acids Res.

[31] Romero A, Garcia P (1991). Initiation of translation at AUC, AUA and AUU codons in Escherichia coli.. FEMS Microbiol Lett.

[32] Nett JH, Kessl J, Wenz T, Trumpower BL (2001). The AUG start codon of the Saccharomyces cerevisiae NFS1 gene can be substituted for by UUG without increased initiation of translation at downstream codons.. Eur J Biochem.

[33] Mulero JJ, Fox TD (1994). Reduced but accurate translation from a mutant AUA initiation codon in the mitochondrial COX2 mRNA of Saccharomyces cerevisiae.. Mol Gen Genet.

[34] Folley LS, Fox TD (1991). Site-directed mutagenesis of a Saccharomyces cerevisiae mitochondrial translation initiation codon.. Genetics.

[35] Liljenstrom H, von Heijne G (1987). Translation rate modification by preferential codon usage: intragenic position effects.. J Theor Biol.

[36] Xia X, Xie Z (2001). DAMBE: Software package for data analysis in molecular biology and evolution.. Journal of Heredity.

[37] Xia X (2001). Data analysis in molecular biology and evolution..

[38] Kondow A, Yokobori S, Ueda T, Watanabe K (1998). Ascidian mitochondrial tRNA(Met) possessing unique structural characteristics.. Nucleosides Nucleotides.

[39] Yokobori S, Ueda T, Feldmaier-Fuchs G, Paabo S, Ueshima R (1999). Complete DNA sequence of the mitochondrial genome of the ascidian Halocynthia roretzi (Chordata, Urochordata).. Genetics.

[40] Gissi C, Iannelli F, Pesole G (2004). Complete mtDNA of Ciona intestinalis reveals extensive gene rearrangement and the presence of an atp8 and an extra trnM gene in ascidians.. J Mol Evol.

[41] Hoffmann RJ, Boore JL, Brown WM (1992). A novel mitochondrial genome organization for the blue mussel, Mytilus edulis.. Genetics.

[42] Yokobori S, Oshima T, Wada H (2005). Complete nucleotide sequence of the mitochondrial genome of Doliolum nationalis with implications for evolution of urochordates.. Mol Phylogenet Evol.

[43] Yokobori S, Watanabe Y, Oshima T (2003). Mitochondrial genome of Ciona savignyi (Urochordata, Ascidiacea, Enterogona): comparison of gene arrangement and tRNA genes with Halocynthia roretzi mitochondrial genome.. J Mol Evol.

[44] Serb JM, Lydeard C (2003). Complete mtDNA sequence of the North American freshwater mussel, Lampsilis ornata (Unionidae): an examination of the evolution and phylogenetic utility of mitochondrial genome organization in Bivalvia (Mollusca).. Mol Biol Evol.

